# Antimicrobial resistance of *Neisseria gonorrhoeae* isolated from patients attending sexually transmitted infection clinics in Urban Hospitals, Lusaka, Zambia

**DOI:** 10.1186/s12879-022-07674-y

**Published:** 2022-08-12

**Authors:** Kelvin L. Sarenje, Owen Ngalamika, Margaret C. Maimbolwa, Amon Siame, Sody M. Munsaka, Geoffrey Kwenda

**Affiliations:** 1 Department of Biomedical Sciences, School of Health Sciences, University of Zambia, P.O. Box 50110, Lusaka, Zambia; 2 Department of Dermato-Venereology, University Teaching Hospital, Lusaka, Zambia; 3 Department of Midwifery Child, and Women’s Health, School of Nursing Sciences, University of Zambia, Lusaka, Zambia; 4 Centre for Infectious Disease Research, Lusaka, Zambia

**Keywords:** *Neisseria gonorrhoeae*, Epsilometer test, Antimicrobial resistance, Zambia

## Abstract

**Background:**

*Neisseria gonorrhoeae*, the causative agent for sexually transmitted infection (STI) gonorrhoea, has emerged with a significant public health impact on acquiring resistance to antimicrobials available for treatment. The resistance of *N. gonorrhoeae* limit treatment options and contributed to high morbidity associated with gonorrhoea. Data on antimicrobial resistance (AMR) profiles in *N. gonorrhoeae* is scares in Zambia. This study aimed to determine the antibiotic susceptibilities in *N. gonorrhoeae* isolates from Lusaka, Zambia.

**Methods:**

A prospective cross-sectional study was conducted on 630 STI patients who presented with urethral or vaginal discharge from 2019 to 2020. Urethral and endocervical secretions were cultured on Modified Thayer Martin agar and incubated at 36 °C ± 1 °C in 5% CO_2_ for 24 h. Identification of *N. gonorrhoeae* isolates was achieved by Gram stain, oxidase, nitrocefin disk, BactiCard *Neisseria*, and Viteck® Compact. The AMR profiles were determined using E-test. Statistical significant was determined by Pearson’s Chi-square test, Mann-Whitney U test, or logistic regression with *p*-values of < 0.05 indicating significance.

**Results:**

A total of 630 patients were recruited of which 46% (290/630) with the median of 29 years and interquartile range (IQR) of 19–39 years were male. The median of the females was 26 years and IQR of 15–37 years. *Neisseria gonorrhoeae* was isolated from 19.4% (122/630) patients of which 72.9% (89/122) were male, with highest prevalence of isolation in the age category of 25–34 years. The prevalence of resistance was high to penicillin (85.2%), tetracycline (68.9%) and ciprofloxacin (59.8%) with MIC_90_ of 32 µg/mL, 8 µg/mL, and 8 µg/mL respectively. The isolates had reduced susceptibility to cefixime (1.6%), spectinomycin (4.9%) and (4.9%) for azithromycin. All isolates were susceptible to ceftriaxone. Risk factors associated with AMR were douching in females (AOR 6.69, 95% CI; 1.11–40.31, *p* = 0.039), female gender (AOR 7.64, 95% CI; 1.11–52.33, *p* = 0.048), HIV-positivity (AOR 26.59, 95% CI; 3.67–192.7, *p* = 0.005), no condom use or unprotected sex (AOR 5.48, 95% CI; 1.17–22.75 *p* = 0.026), sex trading (AOR 4.19, 95% CI; 1.55–11.33, *p* = 0.010), and over-counter treatment of ciprofloxacin (AOR 3.44, 95% CI; 1.17–22.75, *p* = 0.023).

**Conclusion:**

The *N. gonorrhoeae* resistance to penicillin, tetracycline and ciprofloxacin was high necessitating revision of the treatment guidelines. However, no resistance to ceftriaxone was detected. Therefore, monitoring of antibiotic resistance remains critical in Zambia.

**Supplementary Information:**

The online version contains supplementary material available at 10.1186/s12879-022-07674-y.

## Background

Gonorrhoea is a sexually transmitted infection (STI) caused by the bacterium *Neisseria gonorrhoeae*, which remains a major global public health concern because of its capacity to acquire high levels of resistance to antimicrobial agents available for treatment [1, 2]. In the recent decades, *Neisseria gonorrhoeae* has dramatically developed plasmid mediated and/or chromosomally mediated antimicrobial resistance (AMR) leading to the removal of the antimicrobial agent usages as standard first line treatment regimens [3]. The emerging resistance of the superbug to recommended dual therapy of third generation cephalosporins (3GCs) such as ceftriaxone, and macrolide azithromycin threatens to undermine preventive and control measures of gonorrheoa worldwide [4–6].

In 2020, the World Health Organization (WHO) estimated 82.4 million incident global cases of gonorrheoa, and second commonly sexually transmitted disease after *Chlamydia trachomatis* infections among adults of 15–49 years of age [2, 7, 8]. The highest incidence rate of gonorrhoea was found in the African region with 50–100 estimated new infections per 1,000 women and men respectively, every year [9]. In the absence of gonococcal vaccine, novel antimicrobial agents that are effective, affordable and accessible are vital to reduce substantial morbidity and spread of the superbug in the era of untreatable gonorrhoea [10–12].

*Neisseria gonorrhoeae* infects the genital tract in women and men and can also be transmitted from mother to child during delivery and cause infection of the eye of the newborn [13–16]. Complications of cervical gonorrhoea include pelvic inflammatory disease (PID), ectopic pregnancy, infertility, premature rapture of membranes (PROM), preterm birth, low birth weight, spontaneous abortions and neonatal ophthalmia which can progress to blindness in untreated cases of gonorrhoea [8, 17–28]. The dissemination of *N. gonorrhoeae* to extra-genital sites causes endocarditis, septic arthritis and meningitis [29, 30]. In addition to causing complications, gonorrhoea is highly associated with HIV acquisition and transmission by increasing the entry of infective inoculums in co-infections [27, 28, 31–35].

The syndromic case management of sexually transmitted infections (STIs) which had contributed highly to AMR due to empirical treatment was being used in many countries in sub-Sahara Africa [9, 36, 37]. The Zambian standard treatment guidelines recommended the use of single dose of ciprofloxacin in the treatment of gonorrhoea (Ciprofloxacin 500 mg PO stat Plus Doxycycline 100 bd PO X 7/7) [38]. There was scarce information on antimicrobial trends monitoring in *N. gonorrhoeae* for proper selection and use of antimicrobial agents despite resistance been an emerging phenomenon in Zambia. Multidrug-resistant (MDR) *N. gonorrhoeae* was defined as isolates resistant to either 3GCs or azithromycin, plus at least two antimicrobial agents (penicillin, ciprofloxacin, tetracycline, spectinomycin), and extensively drug-resistant (XDR) *N. gonorrhoeae* been resistant to 3GCs and azithromycin, plus at least two antimicrobial agents (penicillin, ciprofloxacin, tetracycline) [39].

The aim of this study was to determine the antimicrobial resistance profile of *Neisseria gonorrhoeae* isolated from patients attending sexually transmitted infection clinics in urban hospitals, Lusaka, Zambia.

## Methods

### Study design and population

A prospective cross-sectional study on 122 *Neisseria gonorrhoeae* isolated from 630 urogenital specimens from patients attending STIs clinics in urban hospitals in Lusaka, Zambia. The urethral and endocervical specimens were collected from patients who presented with a discharge from September, 2019 to August, 2020, and submitted for testing to microbiology laboratory at the University Teaching Hospital. The UTH microbiology laboratory participates in WHO and Southern Africa Development Accreditation Service (SADCAS) programs.

### Data collection

The data was collected by trained nurses, clinical officers and medical doctors through face to face interviews technique using structured questionnaires to collect demographical and clinical factors from consented study participants. The questionnaires were adopted after reviewing different research studies done so far in the region.

### Specimen collection

 Urethral swabs from male participates were collected by inserted a flexible wire 2–3 cm into the urethra and rotated gently before withdrawing. In female participants the speculum was inserted to visualise the cervix and a Dacron swab on a rigid shaft was inserted 2–3 cm into the endocervix and removed with rotation from the endocervical canal. The swabs were immediately put in Amies transport media and transported to UTH microbiology laboratory for testing within 15 min of collection.

## Bacterial culture and identification

Urethral and endocervical secretions were inoculated on Modified Thayer Martin medium and incubated at 36 ºC ± 1 °C in 5% CO_2_ for 24 h, and then the plate was read for growth. Identification of the isolates was achieved by Gram stain (GCC Diagnostics, Flintshire. UK. Lot: 1801), oxidase strips (SIGMA-ALDRICH Co., St. Louis, USA. Lot: BCCD8022), BactiCard *Neisseria* kit (REMEL Inc., Lenexa, KS 66,615 USA. Ref. R21110), and Vitek^®^ Compact using NH ID cards (bioMerieux, Marcy-I’Etoile, France) according to the manufacturer’s protocol. The β-lactamase disk (Mast Group Ltd. Merseyside. UK. Ref. D59) was used to identify penicillinase-producing *Neisseria gonorrhoeae* strains (PPNG), and isolates were regarded as having a high level of resistance to tetracycline (TRNG) with MIC values ≥ 16 µg/mL.

### Antimicrobial susceptibility testing

The minimum inhibitory concentrations (MICs; µg/mL) of ciprofloxacin, ceftriaxone, spectinomycin, azithromycin, penicillin, and tetracycline were determined by E-test (bioMerieux, Marcy-I’Etoile, France), on GC-chocolate with 1% Vitox supplement (Beckton Dickison, France) following the manufacturer’s instructions. The interpretation of MIC dilutions in susceptible (S), intermediate (I) and resistance (R) categories were according to Clinical and Laboratory Standard Institute (CLSI) criteria [40]. The plates were inoculated by dipping a sterile swab into a bacterial cell suspension adjusted to 0.5 McFarland standards using a turbidometer (Oxoid Integrated Technologies Ltd, England). The standardised inoculum was then streaked across the surface of the GC-chocolate agar. The plates were dried at ambient temperature for 5 min before applying the E-test strips and incubated at 36^o^C ± 1^o^ C in 5% CO_2_ for 24 h. The SIR categories for antimicrobial agents in µg/mL were as follows: Ciprofloxacin (CIP) S; ≤0.06, I; 0.12–0.5, R; ≥1, ceftriaxone (CTX) S; ≤ 0.25, R; > 0.25, spectinomycin (SPEC) S; ≤ 32, I; 64, R ≥ 128, cefixime (CFX) S; ≤0.25, R; >0.25, azithromycin (AZT) S; ≤1, R; >1, penicillin (PEN) S; ≤0.06, I; 0.12-1, R; ≥2, and tetracycline (TET) S; ≤0.25, I; 0.12-1, R; ≥2. *Neisseria gonorrhoeae* American Type Culture Collection (ATCC) 49,226 was used as a reference strain and was within the acceptable quality control ranges.

### Statistical analysis

Raw data was cleaned, audited and exported to Stata statistical software version 12.1 (Stata, California, USA) for analysis. Descriptive statistics was reported as frequencies and proportions. The Pearson’s Chi-square test was used to determine the bivariate association between individual categorical variables and the outcome variable. Mann-Whitney U test was used to determine the significant difference in the median age of study participants, and median and interquartile ranges were computed. Univariate and multiple logistic regression was used to assess the association between outcome variable and independent variables; the odds ratio (OR), *p*-values and 95% confidence interval were computed. A *p*-value of < 0.05 was taken as indication of statistical significance.

## Results

A total of 630 participants were recruited of which 54% (340/630) were female. *Neisseria gonorrhoeae* was isolated from 19.4% (122/630) of which 72.9% (89/122) were from male. The gonococcal (GC) infection was much higher in males accounting for (73%), and (54.9%) of the participants with GC infection were married. The highest prevalence of GC infection (47.5%) occurred in the age group of 25–34 years, participants without tertiary education (73.7%), employees (68.9%), men who were circumcised (53.9%), and among men (50.8%) of who used condom during sexual intercourse. The HIV positivity among the participants was (26.2%), traded sex (48.8%), over counter treatment (27%) and (36.4%) of females participants were douching. The majority of GC infection (36.9%) were reported from University Teaching Hospital which was the largest and main referral medical centre in Zambia (Table 1).

**Table 1:** Descriptive characteristics of 122 study participants with gonorrhoeae

Variables	Frequency (n)	Percentage (%)
Age (years)		
15–24	36	29.5
25–34	58	47.5
35–44	20	16.4
45–54	8	6.6
Gender		
Female	33	27.0
Male	89	73.0
Marital status		
Single	55	45.1
Married	67	54.9
Occupation status		
Not working	38	31.1
Working	84	68.9
Education level		
No School	2	1.6
Primary	32	26.2
Secondary	58	47.5
College	20	16.4
University	10	8.2
HIV Status		
Negative	90	73.8
Positive	32	26.2
Condom use		
No	60	49.2
Yes	62	50.8
Over-the-Counter		
No	89	73.0
Yes	33	27.0
Douching		
No	21	63.6
Yes	12	36.4
Traded sex		
No	63	51.6
Yes	59	48.4
Circumcised		
No	41	46.1
Yes	48	53.9
Hospital		
University Teaching Hospital	45	36.9
Chipata Hospital	36	29.5
Kanyama Hospital	41	33.6

**Fig. 1:**
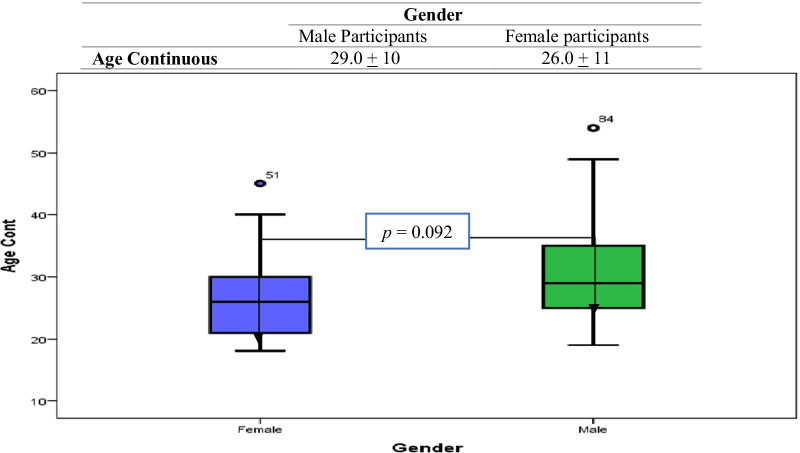
Distribution of age medians for participants with gonorrhoea

The median of age for males was 29 years (interquartile range [IQR], 19 to 39 years), and the median for females was 26 years (IQR, 15–37 years). There was no significant difference in median age between males and females across the categories of gender, *p* = 0.092 (Fig. 1).

Table 2 shows the antimicrobial profile of the 122 *N. gonorrhoeae* isolates, and 85.2% (104/122) isolates were resistant to penicillin with MIC values ranging from 0.047 to 32 µg/mL, MIC_50_ of 32 µg/mL and MIC_90_ of 32 µg/mL, while 68.9% (84/122) isolates were resistant to tetracycline with MIC range of 0.032 to 64 µg/mL, MIC_50_ of 4 µg/mL and MIC_90_ of 8 µg/mL. For ciprofloxacin, 59.8% (73/122) isolates were resistant with MIC values ranging from 0.016 to 32 µg/mL, MIC_50_ of 2 µg/mL and MIC_90_ of 8 µg/mL. Only 1.6% (2/122) isolates were resistant to cefixime with MIC range of 0.016 to 1 µg/mL, MIC_50_ of 0.032 µg/mL and MIC_90_ of 0.19 µg/mL. The resistance of isolates to azithromycin was 4.9% (6/122) with MIC ranging from 0.016 to 4 µg/mL, MIC_50_ of 0.125 µg/mL, and MIC_90_ of 0.75 µg/mL. All isolates were susceptible to ceftriaxone. The PPNG was detected in 45.1%, and TRNG in 2.5%, whereas 1.6% of the isolates presented both phenotypes (PPNG/TRNG).

**Fig. 2:**
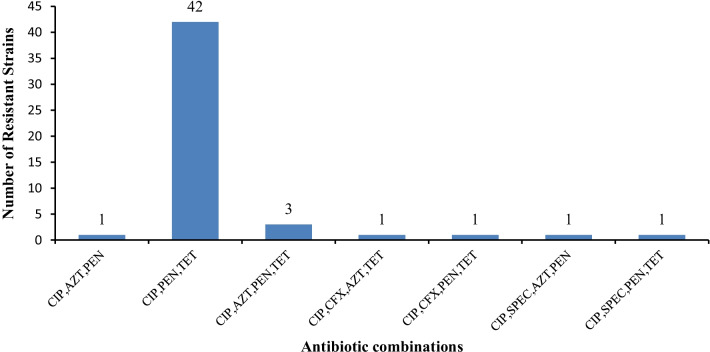
MDR and XDR antibiotic combinations of AMR *N. gonorrhoeae*

Overall, 40.9% (50/122) isolates were resistant to three or more of the tested antimicrobial agents of which 4.9% (6/122) were MDR. Out of six MDR isolates, five were associated with azithromycin resistance and one isolate was associated with cefixime resistance. Only 5.7% (7/122) isolates were resistant to four antimicrobial .agents of which one isolate was XDR showing resistance to cefixime, azithromycin, ciprofloxacin and tetracycline (Fig. 2). The risk factors associated with *N. gonorrhoeae* resistance to ciprofloxacin, tetracycline and penicillin were gender; female (AOR 7.64, 95% CI; 1.11–52.33, *p* = 0.048), HIV-positivity (AOR 26.59, 95% CI; 3.67–192.7, *p* = 0.005), no condom use or unprotected sex (AOR 5.48, 95% CI; 1.17–22.75, *p* = 0.026), sex trading (AOR 4.19, 95% CI; 1.55–11.33, *p* = 0.010) over-counter treatment of ciprofloxacin (AOR 3.44, 95% CI; 1.17–22.75, *p* = 0.023) and douching in females (AOR 6.69, 95% CI; 1.11–40.31, *p* = 0.039) (Additional files 1, 2, 3: Tables S1, S2 and S3)

**Table 2:** In vitro antimicrobial Profile by MIC (µg/mL) using E- test method (n = 122)

	Number of isolate (%)			MIC (µg/mL)		
Antimicrobial Agent	S	I	R	Range	MIC_50_	MIC_90_
Ciprofloxacin	36 (29.5)	12(10.6)	73 (59.8)	0.016-32	2	8
Ceftriaxone	122 (100)	0 (0)	0 (0)	0.016–0.19	0.016	0.125
Spectinomycin	114 (93.4)	2(1.6)	6(4.9)	0.19–192	2	24
Cefixime	120 (98.4)	0(0)	2 (1.6)	0.016-1	0.032	0.19
Azithromycin	116 (95.1)	0(0)	6(4.9)	0.016-4	0.125	0.75
Penicillin G	4 (3.3)	14 (11.5)	104 (85.2)	0.047–32	32	32
Tetracycline	30 (24.5)	08 (6.6)	84 (68.9)	0.032–64	4	8

* S *susceptible, *I* intermediate, *R* resistant, MIC_50_ and MIC_90_: MIC value to inhibit 50% and 90% of the strains tested respectively

## Discussion

Gonococcal infections have been on the increase and could be attributed to different factors in different regions [41]. Data presented in this study showed the highest isolation rate of gonorrhoea in male than female in young adults, and corroborates with findings in South Africa [42]. *Neisseria gonorrhoeae* circulating in Lusaka, Zambia are highly resistant to ciprofloxacin, penicillin and no isolate was resistant to ceftriaxone. The resistant *N. gonorrhoeae* was seven times more likely been isolated from older than younger patients. This could be attributed to vulnerability of the elderly to infections due to weaker immune systems subsequently intensive use of treatment drugs. The female patients are more associated with resistance than men, and those female patients who douche are six times more likely to suffer from AMR than those who were not practicing douching. Statistical association was found between douching and adverse health outcome among Zambian women [43]. HIV positivity, no condom use, and over-counter treatment are others risk factors that showed association with resistant strains of *N. gonorrhoeae*. These results are concordant with findings of the study done in Zimbabwe where HIV positivity, unprotected sex, and self-treatment were associated with high prevalence and resistance of *N. gonorrhoeae* among sexually active age groups [44].

The MDR and XDR *N. gonorrhoeae* showed higher levels of resistance in ciprofloxacin, penicillin and tetracycline as compared to non MDR and XDR *N. gonorrhoeae*. The superbugs showed resistance to cefixime and no resistance to ceftriaxone. *N. gonorrhoeae* strains were highly resistant to penicillin, tetracycline and ciprofloxacin. This is consistent with studies conducted in Europe, and East Asia where resistance to previously recommended treatment such as ciprofloxacin was generally above 50% [10]. According to WHO treatment guidelines the drug of resistance ≥ 5% threshold should not be recommended for treatment [45]. The findings of MDR and XDR pose serious clinical challenges as they indicate the emergence superbugs circulating in the hospitals in Lusaka, Zambia.

## Conclusion

This study showed high prevalence of AMR of *N. gonorrhoeae* associated with various demographics and clinical variables. The *N. gonorrhoeae* showed high resistance to ciprofloxacin, penicillin and tetracycline which poses a treatment challenge. The high level of resistance in ciprofloxacin and tetracycline highlights the need for revision of the national treatment guidelines, and monitoring of antibiotic resistance remains critical in Zambia.

### Limitation of the study

The isolates studied were only from three hospitals in Lusaka and might not be representative of other settings in Zambia.

## Supplementary information

**Additional file 1: TableS1.** Association of demographics and clinicalvariables with *N. gonorrhoeae*resistance to ciprofloxacin.

**Additional file 2: TableS2.** Association of demographics and clinicalvariables with *N. gonorrhoeae*resistance to tetracycline.

**Additional file 3: TableS3.** Association of demographics and clinicalvariables with *N. gonorrhoeae*resistance to penicillin.

## Data Availability

All data generated during the current study are included in this published article and its Additional files.
