# Supplementary material for: Antimicrobial resistance of Neisseria gonorrhoeae isolated from patients attending sexually transmitted infection clinics in Urban Hospitals, Lusaka, Zambia
Source: BMC Infect Dis. 2022 Aug 12;22:688. doi: 10.1186/s12879-022-07674-y (PMC9373640; doi:10.1186/s12879-022-07674-y)
Supplement: Supplementary file 1 — Additional file 1: TableS1. Association of demographics and clinicalvariables with N. gonorrhoeaeresistance to ciprofloxacin. [file 12879_2022_7674_MOESM1_ESM.docx]

**Supplementary Information**

**Tables S1**: Association of demographics and clinical variables with *N. gonorrhoeae* resistance to ciprofloxacin

| Ciprofloxacin Resistance Regression | | | | | | | | | | |
| --- | --- | --- | --- | --- | --- | --- | --- | --- | --- | --- |
|  |  | **Binomial regression** | | | | **Multiple regression** | | | | Variables controlled for: |
|  | Coefficient | p value | COR | 95% C.I. for COR | | p value | AOR | 95% C.I. for AOR | |  |
|  |  |  |  | Lower | Upper |  |  | Lower | Upper |  |
| HIV Status (Susceptible) |  |  |  |  |  |  |  |  |  |  |
| Negative | Ref |  |  |  |  |  |  |  |  | Traded sex, gender and condom use |
| Positive | 1.41 | 0.040 | 4.08 | 1.07 | 15.66 | 0.01 | 7.24 | 1.60 | 32.71 |  |
| Condom use |  |  |  |  |  |  |  |  |  | Traded sex and gender |
| No | 1.66 | 0.026 | 5.24 | 1.22 | 22.42 | 0.031 | 5.48 | 1.17 | 22.75 |  |
| Yes | Ref |  |  |  |  |  |  |  |  |  |
| Over-The-Counter |  |  |  |  |  |  |  |  |  |  |
| No | Ref |  |  |  |  |  |  |  |  |  |
| Yes | 1.23 | 0.023 | 3.43 | 1.19 | 9.89 | 0.022 | 3.44 | 1.19 | 9.93 | Gender |
