# Supplementary material for: Antimicrobial resistance of Neisseria gonorrhoeae isolated from patients attending sexually transmitted infection clinics in Urban Hospitals, Lusaka, Zambia
Source: BMC Infect Dis. 2022 Aug 12;22:688. doi: 10.1186/s12879-022-07674-y (PMC9373640; doi:10.1186/s12879-022-07674-y)
Supplement: Supplementary file 2 — Additional file 2: TableS2. Association of demographics and clinicalvariables with N. gonorrhoeaeresistance to tetracycline. [file 12879_2022_7674_MOESM2_ESM.docx]

**Supplementary Information**

**Tables S2**: Association of demographics and clinical variables with *N. gonorrhoeae* resistance to tetracycline

| Tetracycline Resistance Regression | | | | | | | | | | |
| --- | --- | --- | --- | --- | --- | --- | --- | --- | --- | --- |
|  |  | **Binomial Logistic regression** | | | | **Multiple Logistic regression** | | | | Variables controlled for: |
|  | Coefficient | p value | COR | 95% C.I.for COR | | p value | AOR | 95% C.I.for AOR | |  |
|  |  |  |  | Lower | Upper |  |  | Lower | Upper |  |
| Gender (Intermediate | | | | | | | | | | |
| Females | 1.66 | 0.048 | 5.25 | 1.05 | 26.20 | 0.038 | 7.64 | 1.11 | 52.33 |  |
| Males | Ref |  |  |  |  |  |  |  |  | HIV, Condom use & Trade sex |
| HIV Status (Intermediate) | | | | | | | | | | |
| Negative | Ref |  |  |  |  |  |  |  |  |  |
| Positive | 2.60 | 0.005 | 13.42 | 2.20 | 82.00 | 0.001 | 26.59 | 3.67 | 192.7 | Condom use & Trade sex |
| Condom use (Resistance) | | | | | | | | | | |
| No | Ref |  |  |  |  |  |  |  |  | HIV status & Trade sex |
| Yes | 1.90 | 0.034 | 6.65 | 1.16 | 38.20 | 0.017 | 3.25 | 1.24 | 8.55 |  |
| Trade sex (Resistance) | | | | | | | | | | |
| No | Ref |  |  |  |  |  |  |  |  | HIV status, Condom use & Gender |
| Yes | 1.20 | 0.010 | 3.33 | 1.33 | 8.38 | 0.005 | 4.19 | 1.55 | 11.33 |  |
