# Supplementary material for: Antimicrobial resistance of Neisseria gonorrhoeae isolated from patients attending sexually transmitted infection clinics in Urban Hospitals, Lusaka, Zambia
Source: BMC Infect Dis. 2022 Aug 12;22:688. doi: 10.1186/s12879-022-07674-y (PMC9373640; doi:10.1186/s12879-022-07674-y)
Supplement: Supplementary file 3 — Additional file 3: TableS3. Association of demographics and clinicalvariables with N. gonorrhoeaeresistance to penicillin. [file 12879_2022_7674_MOESM3_ESM.docx]

| Penicillin Resistance Regression | | | | | | | | | |
| --- | --- | --- | --- | --- | --- | --- | --- | --- | --- |
|  |  | **Binomial regression** | | | | **Multiple regression** | | | |
|  | Coefficient | p value | COR | 95% C.I for COR | | p value | AOR | 95% C.I for AOR | |
|  |  |  |  | Lower | Upper |  |  | Lower | Upper |
| Age categories (Years) |  |  |  |  |  |  |  |  |  |
| 15 - 24 | Ref |  |  |  |  |  |  |  |  |
| 25 - 34 | 0.75 | 0.093 | 2.12 | 0.88 | 5.10 |  |  |  |  |
| 35 - 44 | 0.82 | 0.154 | 2.27 | 0.74 | 7.02 |  |  |  |  |
| 45 - 54 | 1.92 | 0.032 | 6.82 | 1.18 | 39.25 | 0.031 | 7.07 | 1.20 | 41.65 |
| Douching(Females) |  |  |  |  |  |  |  |  |  |
| No | Ref |  |  |  |  |  |  |  |  |
| Yes | 1.61 | 0.039 | 5.00 | 1.08 | 23.06 | 0.038 | 6.69 | 1.11 | 40.31 |

**Supplementary Information**

**Tables S3**: Association of demographics and clinical variables with *N. gonorrhoeae* resistance to penicillin
